# 

**Published:** 1878-06-20

**Authors:** 


					﻿M
H
oo
o
h-H
Q
M
05
£
o
o
H
'l ’
05
£
O
*“5
SO
»—I
K
H
O
CO
<«-l
•—I
<1
1-5
Q
M
ffi
H
H
£
W
co
W
05
0^
H
co
<t
cu
iVOL. VIII.	JUNE 20, 1878.	No. 6.
THE SOUTHERN
MEDICAL RECORD.
________\INOEXEd/
fi Monthly j!oui\nal of Practical ^.edicine.
Terms : S3 <>O per annum in advance. Club Itatcs, 5 copies
S8.OO. Single copies 20 cents.
“QUICQl ID PRJECIPIES ESTO BREVIS.”
□2
SB
O
W
H
Q
O
g
!>
h-R
a
•i—i
Q
H
i—i
O
tz!
co
£*
tzj
tJ
•■a
to
f>
o
H
h—<
a
>—<
H
t*
•—
co
co
o
r
o
t—<
H
ESTABLISHED IN 1870.
ATLANTA, GA.: .
Sunny South Steam Publishing House.*
Address R. C. WORD, M. D., Managing Editor, Atlanta, Ga.
				

